# Consumo de tabaco en adultos y cumplimiento de la legislación antitabaco en Costa Rica en 2015

**DOI:** 10.26633/RPSP.2019.42

**Published:** 2019-05-03

**Authors:** Azálea Espinoza Aguirre, Federico Ugalde Montero, Roberto Castro Córdoba, Mónica Quesada Madrigal

**Affiliations:** 1 Dirección de Vigilancia de la Salud Dirección de Vigilancia de la Salud Ministerio de Salud San José Costa Rica Ministerio de Salud, San José, Dirección de Vigilancia de la Salud, Costa Rica.; 2 Departamento de Tecnologías de Información y Comunicación Departamento de Tecnologías de Información y Comunicación Ministerio de Salud San José Costa Rica Ministerio de Salud, Departamento de Tecnologías de Información y Comunicación, San José, Costa Rica.

**Keywords:** Uso de tabaco, adulto, cese del hábito de fumar, tabaco sin humo, legislación como asunto, jurisprudencia, Costa Rica, Tobacco use, adult, smoking cessation, tobacco smokeless, legislation as topic, jurisprudence, Costa Rica, Uso de tabaco, adulto, abandono do hábito de fumar, tabaco sem fumaça, legislação como assunto, jurisprudência, Costa Rica

## Abstract

**Objetivo.:**

Estimar para 2015 el consumo tabaco en adultos y el cumplimiento de las disposiciones incluidas en la Ley General de Control del Tabaco y sus Efectos Nocivos en la Salud y su reglamento.

**Métodos.:**

Se realizó un estudio transversal utilizando la Encuesta Global de Tabaquismo en Adultos (GATS) en hogares, con representación nacional basada en un muestreo probabilístico en tres etapas, por sexo y zona de residencia.

**Resultados.:**

Se visitaron 9 632 hogares donde se realizaron 8 607 entrevistas individuales. La tasa de respuesta fue 89%. Los fumadores actuales fueron 8,9% (IC95%: 8,1-9,8). La prevalencia en hombres fue 13,4% (12,0-15,0), en mujeres, 4,4% (3,7-5,2), en las zonas urbanas, 9,6% (8,5-10,7), en las rurales, 7,1% (6,2-8,2), y la media de la edad de inicio y la de cigarrillos fumados cada día, 16,1 años (15,6-16,6) y 13,4 cigarrillos al día (12,2-14,7), respectivamente. El grupo de 45 a 64 años presentó la mayor prevalencia: 10,4% (9,0-12,0). El 47,5% de los entrevistados nunca había oído hablar de cigarrillos electrónicos, el 6,3% había estado expuesto al humo del tabaco en el lugar de trabajo y 7,9%, en restaurantes.

**Conclusiones.:**

Esta encuesta brindó información política y sanitaria relevante para la vigilancia, la asistencia sanitaria y el control de la legislación antitabaco del país. Debe hacerse un mayor esfuerzo para que se cumplan todas las obligaciones establecidas en la Ley 9028, y los resultados se mantendrán vigentes hasta la segunda encuesta GATS en 2020.

## Abstract (en)

**Objective.:**

Estimate tobacco use in adults in 2015 and observance of the provisions of the General Law for the Control of Tobacco and its Harmful Effects on Health and its regulations.

**Methods.:**

A cross-sectional study was conducted using the Global Adult Tobacco Survey (GATS) in homes, with national representation based on a three-stage probabilistic sample by sex and area of residence.

**Results.:**

Visits were made to 9 632 homes where 8 607 individual interviews were conducted; the response rate was 89%. Current smokers were 8.9% (95% CI: 8.1-9.8). Prevalence in men was 13.4% (12.0-15.0), and in women, 4.4% (3.7-5.2); in urban areas, 9.6% (8.5-10.7), in rural areas, 7.1% (6.2-8.2); and mean age of smoking initiation and mean number of cigarettes smoked per day, 16.1 years (15.6-16.6) and 13.4 (12.2-14.7), respectively. Prevalence was highest in the group aged 45 to 64 years: 10.4% (9.0-12.0); 47.5% of interviewees had never heard of electronic cigarettes; and 6.3% had been exposed to tobacco smoke in the workplace and 7.9% in restaurants.

**Conclusions.:**

This survey yielded policy and health information relevant to surveillance, health care, and enforcement of Costa Rica’s antismoking legislation. Greater effort is needed to ensure that all obligations under Law 9028 are met, and that the results will remain in place until the second GATS in 2020.

## RESUMO

**Objetivo.:**

Estimar para o ano de 2015 o consumo de tabaco em adultos e o cumprimento das disposições contidas na Lei Geral de Controle do Tabaco e dos Efeitos Nocivos à Saúde e da respectiva regulamentação.

**Métodos.:**

Um estudo transversal foi realizado baseado na pesquisa domiciliar Global Adult Tobacco Survey (pesquisa global de consumo de tabaco em adultos, GATS), com representatividade nacional a partir de amostragem probabilística em três etapas, estratificada por sexo e área de residência.

**Resultados.:**

Foram visitados 9.632 domicílios e realizadas 8.607 entrevistas individuais. A taxa de resposta foi de 89%. O percentual de fumantes atuais foi de 8,9% (IC 95% 8,1-9,8). A prevalência do consumo de tabaco no sexo masculino foi de 13,4% (12,0–15,0); no sexo feminino, 4,4% (3,7–5,2); nas áreas urbanas, 9,6% (8,5–10,7) e nas áreas rurais, 7,1% (6,2–8,2). A média da idade de início do consumo de tabaco foi 16,1 anos (15,6–16,6) e a média de consumo foi de 13,4 cigarros ao dia (12,2–14,7). A prevalência maior de consumo de tabaco foi observada em indivíduos de 45 a 64 anos (10,4%; 9,0–12,0). Um percentual de 47,5% dos participantes da pesquisa nunca havia ouvido falar de cigarros eletrônicos e 6,3% haviam sido expostos à fumaça do cigarro no local de trabalho e 7,9%, em restaurantes.

**Conclusões.:**

A pesquisa proporcionou aporte de importância política e de saúde à vigilância, assistência de saúde e controle da legislação antifumo do país. Faz-se necessário um esforço maior para que todas as disposições estabelecidas na Lei 9028 sejam cumpridas. E os resultados continuarão vigentes até a próxima rodada da pesquisa GATS em 2020.

El consumo de tabaco es una de las principales causas prevenibles de muerte y enfermedad prematura en el mundo. De los siete millones de personas que fallecen cada año como consecuencia de su consumo, seis millones son fumadoras y alrededor de 890 000 no, aunque están expuestas al humo del tabaco. El 50% de los consumidores de tabaco pueden morir por cualquiera de las enfermedades que éste ocasiona. Alrededor de 80% de los más de mil millones de fumadores que hay en el mundo viven en países de ingresos bajos o medios (1). Por otro lado, la mortalidad asociada con el consumo de tabaco tiene asociados otros costos, que se relacionan, entre otros factores, con la atención de las enfermedades causadas por el tabaco y la disminución de la productividad de los enfermos (2).

En América Latina, el tabaco ha sido responsable de más de un millón de muertes anuales (3) y muchos países de esta Región padecen esta epidemia, como Chile, donde en 2014 se registró la mayor prevalencia mensual de tabaquismo, 35% (36% en hombres y 34% en mujeres), solo superada por Cuba (4-6).

En Costa Rica, la prevalencia del consumo de tabaco en 2010 en la población entre 12 y 70 años de edad ascendió a 24,8%, 30,7% en los hombres y 18,7% en las mujeres (7). En 2015, el tabaco causó 1 747 muertes en el país, 9,3% del total de fallecimientos de ese año, y 13 718 casos de enfermedad, de los cuales 6 495 correspondieron a infartos y otras enfermedades cardiovasculares, 5 212, a nuevos casos de enfermedad pulmonar obstructiva crónica (EPOC), 500, a accidentes cerebrovasculares y 756, a nuevos casos de cáncer. El costo directo anual fue de ₡129 133 267 938 (colones), lo que equivale a 0,47% del producto interior bruto (PIB) y a 4,8% del gasto anual público en salud (8).

La Organización Mundial de la Salud (OMS) ha propuesto a los países reducir la carga mundial de enfermedades y muertes causadas por el tabaco, promoviendo el compromiso de firmar y ratificar el Convenio Marco de la OMS para el Control del Tabaco (CMTC) (9) y la utilización del paquete de políticas MPOWER, que comprende seis estrategias de control del tabaquismo: (M) Monitoreo del consumo de tabaco y políticas de prevención; (P) Proteger a las personas del humo de tabaco; (O) Ofrecer ayuda para dejar de consumir tabaco; (W) Prevenir los peligros de consumir tabaco; (E) Hacer cumplir las prohibiciones a la publicidad, promoción y patrocinio del tabaco, (R) y Aumentar los impuestos al tabaco (10).

El 23 de julio de 2003, Costa Rica aprobó el CMCT en cada una de sus partes (11) y en agosto de 2008 lo ratificó con la Ley N°8655 (12) y el Decreto Ejecutivo N° 34705 (13). De esta manera, el país inició la aplicación de sus disposiciones a través de leyes, reglamentos y otras medidas para su cumplimiento. En 2012, entró en vigor la Ley General de Control del Tabaco y sus Efectos Nocivos en la Salud (Ley 9028) (13) y su Reglamento (14). A pesar de que Costa Rica contaba con información sobre consumo de tabaco (7), se requería conocer el cumplimiento de las obligaciones del CMCT de la OMS y de los objetivos planteados en la Ley 9028. Por este motivo, las autoridades del Ministerio de Salud (MS) decidieron realizar la encuesta GATS (Global Adults Tobacco Survey) (15), la primera encuesta integral realizada en Costa Rica sobre el consumo y las conductas relacionadas con el tabaco. El objetivo de este estudio es estimar el nivel de consumo tabaco en adultos y el cumplimiento de las disposiciones dadas en la Ley 9028 y su reglamento.

## MATERIALES Y MÉTODOS

Se realizó un estudio transversal con hombres y mujeres de 15 y más años de edad, de zonas urbanas y rurales del país, que vivían en un hogar conforme a la clasificación geográfica indicada por el Instituto Nacional de Estadísticas y Censos del Costa Rica (INEC) (16). Una vez completada la fase de muestreo geográfica, en cada vivienda se seleccionó aleatoriamente una persona adulta para responder a la encuesta.

Se diseñó un muestreo probabilístico en tres etapas: selección proporcional al tamaño de las unidades primarias de muestreo (UPM), selección sistemática de viviendas, y la selección aleatoria de los adultos. La representación a nivel nacional se obtuvo con dos tipos de conglomerados: por sexo y por zona de residencia (urbana y rural). Para la selección de las UPM, se utilizó como marco de referencia el X Censo Nacional de Población y VI de Vivienda, que incluye un total de 10 381 segmentos censales distribuidos en siete provincias, que contienen entre 100 y 125 viviendas (17). Se excluyeron del estudio los siguientes adultos: los recluidos en cárceles y asilos, los enfermos ingresados en hospitales, los que padecen discapacidades mentales y los residentes temporales (con menos de seis meses en el hogar, como estudiantes, obreros y turistas).

El tamaño mínimo de la muestra fue de 8 000 entrevistas. Las viviendas se seleccionaron de manera sistemática: 1 de cada 4 viviendas para visitar aproximadamente 24 viviendas por UPM. En cada unidad de muestreo, se definió un punto conocido de inicio a partir del cual se iba recorriendo el segmento de izquierda a derecha hasta cubrirlo por completo. Los mapas y las listas de las viviendas fueron actualizados por funcionarios del MS. Se capacitaron encuestadores, supervisores y funcionarios del MS utilizando las guías proporcionadas por el Centro para el Control y la Prevención de Enfermedades de Atlanta, Estados Unidos de América (CDC) (18-20).

Para estimar el tamaño de la muestra se tomó como base la prevalencia del consumo actual de tabaco (13,4%) según la Encuesta Nacional de Consumo de Drogas 2010 (7), y se fijaron un error en las estimaciones de 3,0%, un nivel de confianza de 95% y un efecto de diseño de 2,0. La fórmula utilizada fue la siguiente:

$$n=\frac{Z_{1-\text{α}/2}^{2}\cdot P(1-P)\cdot DEFF}{\delta^{2}}$$

donde:

n = tamaño de la muestra

P = prevalencia estimada del consumo de tabaco en 2010

$Z_{1-\text{α/}2}^{2}$ = 95% de la distribución normal estándar de 2

δ = límite para el error de estimación

DEFF = efecto de diseño.

El tamaño de la muestra inicial fue de 2 000 personas para cada conglomerado, sin ajuste por no respuesta para un total de 8 000 personas. La muestra final ajustada con la no respuesta fue de 9 600 individuos. Se visitaron 4 850 viviendas en el área urbana y 4 830 en el área rural. El cálculo de los porcentajes de no respuesta, de tamizaje y de respuesta se realizó a partir del Manual para el Diseño de Muestra, capítulo 10 (21). Se calculó en un 15%, teniendo en cuenta los resultados obtenidos en la encuesta de Indicadores Múltiples por Conglomerados (22) y fue necesario visitar 9 600 viviendas. Se seleccionaron 400 UPM, 200 urbanas y 200 rurales. La distribución de las UPM se hizo según la proporción de habitantes por provincias. La muestra final efectiva fue de 8 607 entrevistas individuales.

El cuestionario empleado fue el del GATS (Global Adult Tobacco Survey Collaborative Group) adaptado a Costa Rica con tabletas electrónicas. Los encuestadores y supervisores fueron capacitados para utilizar el programa informático que contenía el cuestionario (23-25). El personal de tecnologías de la información del MS programó el cuestionario para incluirlo en las tabletas y también recibió capacitación (26). El cuestionario se estructura con 112 preguntas de respuesta dicotómica, de valoración y selección múltiple, e incluye dos compontes: a) el cuestionario del hogar dirigido a conocer las características generales del hogar que se constituye en la base del proceso aleatorio para la selección del entrevistado, y b) el cuestionario individual integrado por un conjunto de preguntas específicas relacionadas con el objeto de estudio. Además, contiene 9 secciones, que cubren las siguientes áreas: características sociodemográficas; consumo de tabaco; tabaco sin humo; cigarrillos electrónicos; cese del consumo; humo de segunda mano; aspectos económicos; medios de comunicación, y conocimientos actitudes y percepciones. Con este cuestionario Costa Rica incorporó por primera vez preguntas sobre el uso del cigarrillo electrónico.

Las variables de este estudio se tomaron de la encuesta GATS y su operacionalización se presenta en el cuadro 1, que incluye la definición, las categorías, el indicador y la medición de cada una de ellas. Dichas variables se agruparon mediante los siguientes indicadores: sociodemográficos, económicos, de vigilancia para el monitoreo del consumo de tabaco, y de cumplimiento de las disposiciones dadas en la Ley 9028. En el grupo de indicadores sociodemográficos se incluyeron las variables sexo, edad, nivel educativo y zona de residencia. El nivel de educación consta de las siguientes categorías: menos que primaria, primaria completa, menos que secundaria completa, escuela secundaria completa, universidad completa, grado de postgrado, grado de postgrado completo, no sabe y rechaza responder.

**CUADRO 1:** Definición, categorías, indicador y medida de las variables del estudio, Costa Rica, 2015

Variables	Definición	Categorías	Indicador	Medida
Consumo de tabaco	Adultos (de 15 años y más, hombres y mujeres de zona urbana y rural) que consumen tabaco en cualquiera de sus formas: fumado, inhalado y mascado (consumo actual se refiere a consumo diario y menor que en los últimos 30 días)	Fumadores de tabaco Fumadores actuales: fumadores diarios y fumadores ocasionales	Vigilancia	Prevalencia
	Adultos que fuman diariamente	Fumadores diarios		
	Adultos que consumen tabaco en forma de cigarrillos. Incluye cigarrillos manufacturados y cigarrillos hechos a mano	Fumadores actuales de cigarrillos		
	Adultos que consumen tabaco diariamente	Fumadores diarios de cigarrillos		
	Edad en años en que los adultos empezaron a fumar	Adultos	Vigilancia	Media
	Entre los adultos que fuman cigarrillos diariamente	Promedio de cigarrillos fumados a diario	Vigilancia	Media
Cesación	Adultos fumadores actuales y los que dejaron de fumar en los últimos 12 meses	Fumadores que hicieron un intento de dejar de fumar en los últimos 12 meses	Cumplimiento de la Ley 9028	Porcentaje
		Fumadores actuales que planearon o están pensando dejar de fumar		
		Fumadores que fueron aconsejados a dejar de fumar por un médico o proveedor de salud en los últimos 12 meses		
Exposición al humo de segunda mano	Adultos entre los que trabajan fuera del hogar en espacios cerrados, abiertos o en ambos	Adultos expuestos al humo de tabaco en su lugar de trabajo	Cumplimiento de la Ley 9028	Porcentaje
		Adultos expuestos al humo de tabaco en bares o clubes nocturnos		
		Adultos expuestos al humo de tabaco en restaurantes		
		Expuestos al humo de tabaco en su hogar en el último mes		
Economía	Gasto en colones (moneda de Costa Rica) mensual de los adultos entre los que consumen cigarrillos	Gasto medio (colones) en cigarrillos por mes	Económico	Media
Medios de comunicación	Adultos que vieron los precios de venta de los cigarros, regalías u ofertas de descuento en otros productos cuando compraron cigarros o alguna publicidad o señales de promoción de cigarros en los sitios de venta de cigarros durante los últimos 30 días	En tiendas de venta de cigarros. Incluye fumadores y no fumadores	Cumplimiento de la Ley 9028	Porcentaje
		Adultos que observaron publicidad o promoción de cigarrillos en eventos deportivos		
	Vieron publicidad en contra durante los últimos 30 días	Fumadores que pensaron dejar de fumar por las advertencias en paquetes de cigarrillos durante los últimos 30 días	Cumplimiento de la Ley 9028	Porcentaje
		Adultos que vieron información en contra de fumar en la televisión o la escucharon por radio		
Conocimientos, actitudes y percepciones	Adultos entre fumadores y no fumadores que expresaron sus conocimientos, actitudes y percepciones sobre los daños que produce el tabaco en la salud de las personas	Adultos que creen que el fumar causa enfermedades graves	Indicador de conocimiento	Porcentaje
	Adultos entre fumadores y no fumadores que expresaron sus conocimientos, actitudes y percepciones sobre los impuestos a los productos de tabaco	Adultos a favor del aumento de impuestos a los productos de tabaco		
		Adultos a favor de leyes que prohíben fumar en lugares públicos		
Cigarrillos electrónicos	Sistema electrónico para consumir tabaco. Utiliza una batería, que calienta una solución líquida para convertirla en vapor. Su diseño generalmente imita un cigarrillo o una pipa	Adultos consumidores actuales de cigarrillos electrónicos	Cumplimiento de las disposiciones de la Ley 9028	Porcentaje

*Fuente*: cuestionario del GATS adaptado a Costa Rica (18-21).

Se realizó una prueba piloto del 23 al 27 de febrero de 2015, supervisada por personal del MS, de la Organización Panamericana de la Salud (OPS) y del CDC. Las estimaciones, la ponderación y los intervalos de confianza de la muestra se calcularon utilizando el módulo para muestras complejas del paquete estadístico SPSS 23. Las tablas y gráficos empleados se construyeron en Excel 2013 (21, 27). Se estimaron medias y prevalencias del consumo de tabaco y sus correspondientes intervalos de confianza de 95% (IC95%).

El cuestionario individual contenía un consentimiento informado digital, donde se incluyeron todos los principios éticos fundamentales de la investigación. Para los menores de 18 años, se diseñaron los consentimientos para sus padres o encargados y asentimientos para los menores. Los consentimientos y los asentimientos estuvieron siempre disponibles de forma impresa y en formatos electrónicos en la tableta de los entrevistadores (24). Como esta investigación fue realizada por el MS de Costa Rica, es de tipo observacional y de interés nacional, y según lo estipulado en el Artículo 7 de la Ley Reguladora de Investigación Biomédica, Ley 9234 de Costa Rica, no fue necesario someterla a la aprobación del Comité Ético Científico.

## RESULTADOS

Las características sociodemográficas de los integrantes de la muestra se presentan en el cuadro 2. Se elaboraron una hoja resumen y un resumen ejecutivo para la divulgación de los resultados preliminares de la encuesta (28, 29). Como la investigación se realizó con representación nacional, los resultados pueden generalizarse a toda la población.

**CUADRO 2:** Porcentaje y distribución de los adultos por características demográficas seleccionadas según la muestra ponderada y número de adultos sin ajustar, Costa Rica, 2015

Características demográficas		Muestra ponderada			Número de adultos sin ajustar
		Porcentaje (IC95%)		Número de adultos (en miles)	
General			100	3 655,2	8 607
Sexo					
	Masculino	50,3	(48,9-51,7)	1 837,1	3 544
	Femenino	49,7	(48,3-51,1)	1 818,1	5 063
Edad (en años)					
	15-24	23,3	(21,9-24,7)	852,1	1 377
	25-44	40,9	(39,4-42,4)	1 494,8	3 049
	45-64	26,5	(25,2-27,7)	967,2	2 662
	65+	9,3	(8,6-10,1)	341,1	1 519
Residencia					
	Urbana	74	(73,2-74,7)	2 703,4	4 257
	Rural	26	(25,3-26,8)	951,8	4 350
Nivel de educación					
	Menos de primaria	12,6	(11,7-13,6)	457,2	1 687
	Primaria completa	53,1	(51,3-54,9)	1 927,8	4 577
	Secundaria completa	28,9	(27,0-30,9)	1 049	1 931
	Universidad o más	5,4	(4,4-6,6)	196,7	375

IC95%: intervalo de confianza de 95%.

*Fuente*: Datos de la propia investigación y del Instituto Nacional de Estadísticas y Censos de Costa Rica.

**CUADRO 3:** Porcentaje de la población que notificó incumplimientos en las disposiciones de la Ley 9028, Costa Rica, 2015

Disposiciones de la Ley 9028			Porcentaje	IC95%
Artículo 5. Protección contra el humo del humo de tabaco				
	Exposición al humo de segunda mano en el trabajo		6,3	5,1-7,8
	Exposición al humo de segunda mano en el hogar		4,9	4,2-5,7
	Exposición al humo de segunda mano en lugares públicos:			
		Bares/Clubes nocturnos	23	20,2-26,2
		Universidades	17,7	14,3-21,7
		Restaurantes	7,9	6,7-9,4
Artículo 9. Etiquetado de los productos de tabaco para prevenir sobre los peligros de consumir tabaco				
	Cree que fumar tabaco causa enfermedades graves		97,8	97,3-98,2
	Cree que fumar causa:			
		Derrame cerebral	71,2	69,9-72,6
		Infarto del miocardio	90,4	88,3-91,7
		Cáncer de pulmón	98,2	97,8-99,3
		Bronquitis crónica	94,8	94,1-95,4
Artículo 12. Hacer cumplir las prohibiciones a la publicidad, promoción y patrocinio del tabaco				
	Vio cualquier publicidad, promoción y patrocinio de cigarrillos		27,4	25,3-29,7
Artículo 16. Regulación de la venta y suministro de productos de tabaco en determinados lugares y espacios				
		Hospitales	99,2	98,8-99,4
		Lugares de trabajo	98,5	98,1-98,8
		Bares	94,6	93,8-95,3
		Transporte público	99,1	98,8-99,3
		Centros educativos	94,4	99,1-96,6
		Universidades	98,9	98,5-99,1
		Lugares de culto	99,1	98,7-99,4
		Restaurantes	98,6	98,1-98,9
Artículo 18. Regulación del comercio, distribución y venta de productos de tabaco				
	Fuente de la última compra de cigarrillos:			
		Establecimientos comerciales	80,7	76,0-84,6
		Tiendas libres de impuestos	6,2	4,3-8,9
		Vendedores callejeros	5,4	3,4-8,5
Artículo 22. Impuesto a los productos de tabaco				
Estuvo de acuerdo en aplicar la ley en lugares públicos			93,1	92,2-93,8
Artículo 4. Reglamento de la Ley 9028: Cigarrillos electrónicos				
	Adultos que no escuchado sobre cigarrillos electrónicos		47,5	45,6-49,4
	Consumidores actuales de cigarrillos electrónicos		1,3	1,0-1,7

IC95%: intervalo de confianza de 95%.

*Fuente*: Datos de la investigación.

La prevalencia general del consumo de tabaco (fumado y tabaco sin humo) en los adultos fue 9,1% (IC95%: 8,2-10,0). En los hombres el porcentaje fue más alto que en las mujeres (13,6%; IC95%: 12,2-15,2 y 4,5%: IC95%: 3,8-5,2, respectivamente).

En los adultos fumadores de tabaco la prevalencia fue 8,9% (8,1-9,8), 13,4% (12,0-15,0) en los hombres y 4,4% (3,7-5,2) en las mujeres. De ellos, 5,8% (5,2-6,6) fueron fumadores diarios, 8,7% (7,5-10,0) hombres y 2,9% (2,4-3,6) mujeres. La media de edad de inicio de los fumadores diarios en el total de la población fue 16,1 años (15,6-16,6) y la media de cigarrillos fumados cada día, 13,4 (12,2-14,7) en la población total. La prevalencia de consumo fue más alta en zonas urbanas (9,6%; 8,5-10,7) que en las rurales (7,1%; 6,2-8,2).

**FIGURA 1:**
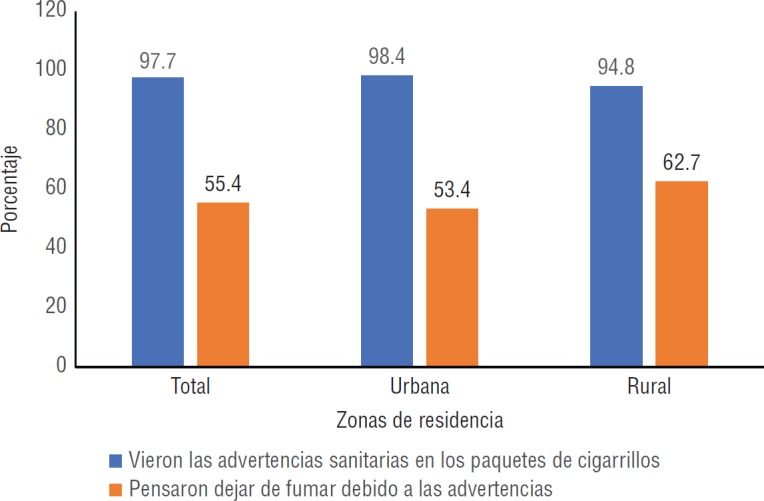
Porcentaje de fumadores que vieron las advertencias sanitarias en los paquetes de cigarrillos y consideraron dejar de fumar por las etiquetas de advertencia durante el último mes según su lugar de residencia, Costa Rica, 2015

*Fuente*: Elaboración propia.

Casi 6 de cada 10 fumadores del año anterior (58,6%; 53,7-63,2) intentaron dejar de fumar en los últimos 12 meses. Entre los que intentaron dejar de fumar, 3,6% (2,1-6,4) trataron de hacerlo usando métodos de farmacoterapia, 6,7% (4,3-10,2) usaron consejería o asesoramiento, y 64,7% (58,5-70,5) lo hicieron sin ninguna ayuda. De los fumadores del año anterior que visitaron a un proveedor de servicios de salud en los últimos 12 meses, solo a 64,0% (56,3-71,0) se les recomendó dejar de fumar. El 73% (71,8-74,2) de los fumadores actuales planeaban o estaban pensando dejar de fumar.

En el cuadro 2 aparecen los indicadores del monitoreo del cumplimiento de la Ley 9028.

En cuanto al análisis económico, se estimó que el gasto medio por mes en cigarrillos manufacturados de los fumadores de cigarrillos fue ₡19 370, y el gasto de consumo de tabaco respecto al ingreso total del hogar, 1,3%. El gasto medio en 20 cigarrillos manufacturados fue ₡1 328 y el costo de 100 paquetes (o 2 000 cigarrillos) de cigarrillos manufacturados como porcentaje del PIB per cápita de 2014, 2,4% (30).

En la figura 1 se presentan los resultados de la publicidad en medios de comunicación. Con respecto a los cigarrillos electrónicos, 47,5% del total de entrevistados nunca escuchó hablar de ellos y el porcentaje de consumidores fue 1,3%.

## DISCUSIÓN

Los principales resultados de este estudio muestran cambios en la prevalencia de consumo actual de tabaco en adultos respecto a encuestas anteriores, deficiencias del MS en hacer cumplir la Ley antitabaco y, específicamente, en proteger a la población de la exposición pasiva al humo y de la publicidad de la industria tabacalera, así como debilidades en los centros asistenciales que ofrecen ayuda para dejar de fumar.

El último estudio nacional realizado mostró que la prevalencia de consumo de tabaco durante el último mes fue 14,3% y que había descendido el consumo en los adultos (7). Asimismo, el indicador consumo actual de tabaco, obtenido por el GATS en 2015, fue 8,9%. Ocurrió lo mismo en las mujeres: el consumo disminuyó, pasando de 8,6% a 4,5% en 2010 (7). Es posible que las medidas de regulatorias adoptadas en el país para el control de tabaco, como incrementar los impuestos a las cajetillas de cigarrillos y educar a la población sobre los efectos perjudiciales del tabaco para la salud, hayan sido exitosas al desmotivar el consumo de tabaco (1, 5).

La encuesta GATS de Costa Rica pone de relieve incumplimientos de la legislación. Aunque el Artículo 5 establece que la protección contra el humo de tabaco en el lugar de trabajo, en restaurantes y en el transporte público debe ser 100%, un 6,3% de la población está desprotegida. El MS debe hacer cumplir la Ley a través de la Policía Nacional, de las municipalidades y la denuncia ciudadana, como se indica en los Artículos 36, 43 y 48 (13, 14).

En el Artículo 7, relacionado con el cese del hábito tabáquico, se otorga a los consumidores permisos para que puedan acudir a las clínicas oficiales de cesación; sin embargo, 58,6% de los fumadores que intentaron dejar de fumar en el último año (2014) y no lo lograron, dejaron de acudir. Probablemente los profesionales de la salud del país deban adquirir capacitación y destrezas para ayudar a los pacientes a dejar de fumar. El abandono de la adicción podría superar la capacidad de la acción individual y debería abordarse como un problema de salud pública y acompañarse de intervenciones novedosas dirigidas a los consumidores, como el apoyo en el hogar y en la comunidad, que no se ofrecen en las clínicas de cesación de Costa Rica (31-33). El MS y la Seguridad Social pueden readecuar la oferta de recursos para promover el cese del tabaquismo.

El uso del cigarrillo electrónico en Costa Rica fue bajo (1,3%). No obstante, se ha convertido en moda, se vende como un medio para reducir el consumo de tabaco y algunos consideran que podría ayudar a reducir el esfuerzo de los países para disminuir el consumo, si bien el MS debería regular su uso (34).

La publicidad, la promoción y el patrocinio de productos del tabaco están prohibidos por el Artículo 12, pero 15% de la población identifica la publicidad. Las advertencias sanitarias gráficas en las dos caras principales de los paquetes de cigarrillos han contribuido a que más de 50% de los fumadores dejaran de fumar (35). Por ello, el MS debe eliminar la publicidad y avanzar en la implementación del etiquetado plano.

Los indicadores obtenidos con la encuesta GATS han permitido al MS obtener una visión amplia de la situación del tabaquismo en Costa Rica respecto a otros estudios realizados en el país, que solo han notificado prevalencias en la población general por sexo y grupos de edad (7). Esta es la primera vez que se consigue obtener información sobre el tabaquismo, las conductas relacionadas con el tabaco y la legislación a escala nacional y comparable con la de otros países.

Como principal limitación de este estudio cabe señalar que no fue posible realizar la encuesta en una de las UPM rurales por problemas de accesibilidad, lo que no afectó la representatividad de la muestra final ni los resultados obtenidos.

Como conclusiones deben destacarse las siguientes. Primera, la encuesta GATS de Costa Rica de 2015 brindó información de relevancia política y sanitaria, habida cuenta de que ha dado a conocer aspectos clave para la vigilancia, la asistencia sanitaria y el control de la legislación antitabaco del país. Por ello, este estudio puede ser un referente para los que se realicen en el futuro. Segunda, Costa Rica ha puesto en marcha políticas de control del tabaco que han demostrado ser exitosas a juzgar por los resultados de esta investigación. Sin embargo, las autoridades de salud y la policía nacional deben hacer un mayor esfuerzo para que se cumplan todas las obligaciones establecidas en la Ley 9028. Y tercera, a pesar de que la investigación se realizó en 2015, hasta la fecha Costa Rica no dispone de ningún otro estudio, por lo que se mantendrá vigente hasta que se realice la segunda encuesta GATS en 2020.

## Contribución de los autores.

Todos los autores han participado en el diseño del estudio original, en la recolección y análisis de los datos, en la interpretación de los resultados, y en la redacción, revisión y aprobación del manuscrito final.

## Agradecimientos.

Los autores agradecen al Dr. Henry Wasserman la revisión que realizó de este manuscrito.

## Financiación.

Este estudio ha recibido financiación de la Oficina de Control de Tabaco del MS, de la OPS, de los Centros para el Control y la Prevención Enfermedades de Estados Unidos, y de la CDC Foundation. Ninguno de estas organizaciones ha participado en ninguna de las etapas de este estudio ni en la elaboración de este manuscrito.

## Declaración.

Las opiniones expresadas en este manuscrito son responsabilidad del autor y no reflejan necesariamente los criterios ni la política de la RPSP/ PAJPH y/o de la OPS.
